# Gender-Dimorphic Impact of *PXR* Genotype and Haplotype on Hepatotoxicity During Antituberculosis Treatment

**DOI:** 10.1097/MD.0000000000000982

**Published:** 2015-06-19

**Authors:** Jann Yuan Wang, Ching Hui Tsai, Yungling Leo Lee, Li Na Lee, Chia Lin Hsu, Hsiu Ching Chang, Jong Ming Chen, Cheng An Hsu, Chong Jen Yu, Pan Chyr Yang

**Affiliations:** From the Department of Internal Medicine, National Taiwan University Hospital (JYW, CLH, CJY, PCY); Institute of Epidemiology and Preventive Medicine, College of Public Health, National Taiwan University (CHT, YLL); and Department of Laboratory Medicine, National Taiwan University Hospital, Zhongzheng District, Taipei, Taiwan (LNL, HCC, JMC, CAH).

## Abstract

Women have a higher risk of drug-induced hepatotoxicity during antituberculosis treatment (HATT) than men. We hypothesized that single nucleotide polymorphism (SNP) genotype and derived haplotype of *pregnane X receptor* (*PXR*) gene, which could regulate the expression of phase I enzyme cytochrome P450 (CYP) 3A4, had a sex-specific influence on the risk of HATT.

Six SNPs of the *PXR* gene were sequenced. Genotypes and haplotypes of the *PXR* SNPs, and other potential risk factors for HATT were compared between pulmonary TB patients with and those without HATT. HATT was defined as an increase in serum transaminase level >3 times the upper limit of normal (ULN) with symptoms, or >5 times ULN without symptoms. We performed the study in a derivation and a validation cohort.

Among the 355 patients with pulmonary TB in the derivation cohort, 70 (19.7%) developed HATT. Logistic regression analysis revealed the risk of HATT increased in female genotype AA at rs2461823 (OR: 6.87 [2.55–18.52]) and decreased in female genotype AA at rs7643645 (OR: 0.14 [0.02–1.02]) of *PXR* gene. Haplotype analysis showed that female h001101 (OR: 2.30 [1.22–4.32]) and female h000110 (OR: 2.25 [1.08–4.69]) haplotype were associated with increased HATT risk. The identified predictors were also significantly associated with female HATT risk among the 182 patients in the validation cohort.

Two *PXR* SNP genotypes and 2 haplotypes influenced the risk of HATT only in females. The *PXR* SNP showed a sex-specific impact that contributed to an increased HATT risk in females.

## Abstract 2

Supplemental Digital Content is available in the text

## INTRODUCTION

Tuberculosis (TB) remains a major infectious cause of deaths worldwide. To prevent transmission and future relapse, prompt and supervised anti-TB treatment for an extended period is very important.^1^ However, the development of hepatotoxicity, which may be induced by anti-TB drugs or acute flare-up of concomitant viral hepatitis, is the most important adverse event leading to interruption or premature discontinuation of anti-TB treatment.^2^ Among the first-line anti-TB drugs, isoniazid (INH), rifampin (RMP), and pyrazinamide (PZA) are all hepatotoxic and can increase the risk of hepatotoxicity further when used together.

For a long time, women have been reported to have a higher risk of hepatitis during antituberculosis treatment (HATT) than men. Although the definition of hepatotoxicity varies between studies, the hazard ratio of female sex ranges from 1.5 to 3.3.^3–7^ A previous study in Taiwan revealed that female sex was a significant risk factor of drug-induced HATT and was independent of the *N-acetyltransferase 2* (*NAT2*) status.^6^ One possible reason is the activity of cytochrome P450 (CYP) 3A4, the most abundant enzyme in the hepatic CYP family that catalyzes the phase I reaction of many drugs, is higher in women,^8^ given that many adverse drug reactions are caused by the CYP dependent activation of drugs into reactive metabolites.^9^

The exact mechanism that leads to higher CYP3A4 activity in women than men is unknown. Single nucleotide polymorphisms (SNPs) in the coding region of the *CYP3A4* gene occur only rarely and cannot explain the difference in CYP3A4 activity between men and women.^10^ The pregnane X receptor (PXR), a member of the nuclear receptor superfamily, is a known regulator of the *CYP3A4* gene. When bound by its ligand, PXR upregulates the expression of its target genes, including genes for phase 1 metabolizing enzymes such as CYP3A, and genes for all 3 phases of xenobiotic metabolism.^11,12^ SNPs in the transcription factor binding sites of the *PXR* regulatory region (the promoter and intron1) have also been associated with altered *PXR* and *CYP3A4* expressions,^13,14^ as well as drug-induced liver injury.^15^

Given the large spectrum of PXR activating ligands and target genes, and the association between SNPs in the *PXR* regulatory region and *CYP3A4* expression, it is possible that gene variants in the *PXR* regulatory region may contribute to differences in risk of drug-induced HATT between male and female patients. We hypothesized that certain genotypes and haplotypes in *PXR* regulatory region SNPs may be risk factors for HATT, and the distribution of these genotypes and haplotypes may be different between male and female TB patients, leading to the increased risk of hepatotoxicity in females.

## METHODS

### Study Population and Protocols

This prospective study was conducted at National Taiwan University Hospital, a tertiary-care center in Taiwan. The hospital's Institution Review Board approved the study (NTUH REB No.: 9561707008). All of the participants provided informed written consent.

From March 2007 to February 2010, adult patients (>16 years) with culture-confirmed pulmonary TB were enrolled as the derivation cohort. Mycobacterial culture and drug susceptibility testing were performed as previously described.^16^ Subjects were excluded if they were pregnant, had a life expectancy <6 months, had abnormal baseline liver function test (LFT), or had *Mycobacterium tuberculosis* (MTB) isolates resistant to INH, RMP, or both. From March 2010 to February 2013, TB patients fulfilling these criteria were enrolled as the validation cohort.

Complete medical data and radiologic imaging were recorded. Alcohol abuse was defined as a daily consumption of ≥60 g of alcohol.^17^ Malnutrition was defined as either serum albumin level <3.5 g/dL^6^ or body-mass index <18.5 kg/m^2^.^18^

For TB patients, LFT, including aspartate transaminase (AST) and alanine transaminase (ALT), direct and total bilirubin levels, were determined before the start of anti-TB treatment. Serologic tests for hepatitis B virus (HBV), hepatitis C virus (HCV), and human immunodeficiency virus, serum albumin, creatinine, and complete hemogram were also performed. The LFT was checked at 2, 4, 6, 8, 12, and 16 weeks after the start of anti-TB treatment or whenever symptoms of hepatitis developed during the initial 6 months of anti-TB treatment.^19^ If there was elevated AST or ALT, the LFT was repeated and assessed weekly. For patients with concomitant HBV or HCV infection, serum HBV or HCV viral load was determined by quantitative PCR (Cobas Amplicor HBV and HCV monitor v2.0; Roche Diagnostics, Pleasanton, CA) simultaneously with LFT to document acute flare-up of viral hepatitis.

All TB patients received standard anti-TB treatment of daily INH, RMP, ethambutol (EMB), and PZA in the first 2 months, and daily INH and RMP for the next 4 months. The daily dosage of each drug was calculated by weight.^20^ The regimen was modified by the primary care physician if necessary, for example, when there were adverse drug effects.

### Definition and Etiology of Hepatitis During Antituberculosis Treatment (HATT)

HATT was defined as increased serum AST and/or ALT >3 times the upper limit of normal (ULN) in symptomatic patients, or >5 times the ULN in asymptomatic patients.^2^ Once HATT occurred, potentially hepatotoxic drugs (INH, RMP, and PZA) were stopped. Anti-TB drugs were reintroduced after serum levels of AST and/or ALT returned to <3 times the ULN and the clinical symptoms of hepatitis resolved. As in a previous study,^6^ the diagnosis of INH- or RMP-induced hepatitis required a positive rechallenge test (at least doubling of serum AST or ALT levels and recurrence of clinical symptoms of hepatitis after rechallenge), whereas PZA-induced hepatitis was diagnosed either by a positive rechallenge test or by exclusion. Virus-induced HATT was diagnosed if the rise in serum AST and ALT was associated with a concomitant rise in viral load.

### Genotyping for *PXR* SNPs

Genotyping for the *PXR* and *NAT2* genes for TB patients was performed on genomic DNA extracted from peripheral white blood cells. Laboratory technicians were blinded to the status of the participants during the entire process of *PXR* and *NAT2* genotyping.

The *PXR* gene, also known as the nuclear receptor subfamily 1, group I, member 2 (*NR1I2*) gene, is located in chromosome 3. We first obtained a list of *PXR* SNPs based on predicted regulatory function or known association with diseases.^13,21–23^ All SNPs were used as input files for the Haploview v4.1 (http://www.broadinstitute.org/mpg/haploview) to search for tag SNPs in the genomic region of *PXR* that encompasses 36 kb and contains 33 polymorphic sites, using squared correlation (*r*^2^) cutoff ≥0.95 and minor allele frequency (MAF) ≥0.1. We selected 6 tag SNPs in the regulatory region of *PXR* gene to be investigated: rs3814055 (located in the 5′ untranslated region), rs12488820, rs2461823, rs7643645 (all located in intron 1), rs6785049 (located in intron 5), and rs3814057 (located in the 3′ untranslated region) (Supplementary Figure S1, http://links.lww.com/MD/A303).

SNP genotyping using the Sequenom MassARRAY system (iPLEX GOLD) (Sequenom, San Diego, CA) was performed according to the manufacturer's recommendations (Sequenom) (see Supplement File for details, http://links.lww.com/MD/A303).^24^ Call rates for individual polymorphisms were >98%. Concordance of duplicates was 100%.

### Genotyping for *NAT2* Gene

Genotyping for *NAT2* was performed by direct sequencing.^6^ Four *NAT2* variants, that is, 191G>A (rs1801279, R64Q or *NAT2*^∗^14 allele), 341T>C (rs180128012, I114T or *NAT2*^∗^5 allele), 590G>A (rs1799930, R197Q or *NAT2*^∗^6 allele), and 857G>A (rs1799931, G286E or *NAT2*^∗^7 allele), result in amino acid substitution and are associated with slow acetylator phenotype. The presence of 2 of these variants in a patient was defined as slow acetylator genotype.

### Data Analysis and Statistical Analysis

All SNPs were tested for Hardy-Weinberg equilibrium.^25^ Double data entry were performed to ensure data quality. Differences between groups were analyzed by independent sampled *t* test for continuous variables and by chi-square test or Fisher exact test for categorical variables. Linkage disequilibrium analysis was performed using Haploview.^26^ Haplotype frequencies were calculated from *PXR* genotype data and analyzed using the EM algorithm by TagSNPs.^27^ We collapsed rare haplotypes (frequency <0.05) into a category in final haplotype analyses.

The association between drug-induced HATT and clinical factors, *NAT2* and *PXR* genotype frequencies, *PXR* allele frequencies, and *PXR* haplotype frequencies were analyzed using chi-square method, univariate, and multivariate logistic regression model. In the multivariate logistic regression analysis, an interaction variable between sex and *PXR* genotypes and haplotypes was also included. The identified predictors of drug-induced HATT were then validated using a validation cohort. A 2-sided *p* < 0.05 was considered significant. All analyses were performed using the SAS (Version 9.2, SAS Institute Inc., Cary, NC).

## RESULTS

### Derivation Cohort Case Enrollment

From March 2007 to February 2010 a total of 964 cases of culture-confirmed pulmonary TB were identified. Of the 964 cases, 222 cases were excluded due to the following reasons: 21 cases with multidrug-resistant MTB isolates which were resistant to both INH and RMP, 104 cases with INH-resistant MTB isolates, 3 cases with RMP-resistant MTB isolates, 51 cases with a life expectancy <6 months, 10 cases with abnormal baseline LFT (7 due to congestive heart failure and 3 due to excessive alcohol consumption), and 33 cases withdrew their consent later. Of the remaining 742 TB patients, 355 (36.8% of 964) agreed to participate, completed the study and formed the derivation cohort. Their mean age was 57.6 ± 19.4 years and 233 (65.6%) were male. The overall follow-up duration was 2004.5 person-months (5.6 months/patient).

### Characteristics of Patients With HATT

During the 6-month follow-up, 70 (19.7%) patients developed drug-induced HATT, including 31 women (25.4% of 122 women) and 39 men (16.7% of 233 men). Sixty (16.9% of 355) patients were symptomatic with transaminases >3 times ULN and the other 10 (2.8% of 355) were asymptomatic with transaminases >5 times ULN. Of the 70 patients with drug-induced HATT, 13 (19% of 70 patients) had serum total bilirubin >2.0 mg/dL (mean 3.3 ± 1.9, range 2.0–7.4 mg/dL). None was complicated with hepatic failure. The responsible anti-TB drug was INH in 8 (11%), RMP in 18 (26%), and PZA in 44 (63%). Thirty three (47%) developed drug-induced hepatitis within the first month of the treatment and 32 (46%) in the second month.

Table 1 shows that when compared to patients without drug-induced HATT, those who developed drug-induced HATT showed a tendency of higher percentage of female gender (44.3% vs 31.9%, *p* = 0.051), *NAT2* slow acetylator genotype (32.9% vs 21.8%, *p* = 0.051, power = 0.489), lower percentage of cavitation on initial chest radiography (7.1% vs 15.8%, *p* = 0.062), and significantly lower probability of positive sputum culture for *M. tuberculosis* after 2 months of anti-TB treatment (0% vs 11.1%, *p* = 0.014). None of the 4 *NAT2* variants (191G>A, 341T>C, 590G>A, and 857G>A) or 7 alleles (^∗^4, ^∗^5, ^∗^6, ^∗^7, ^∗^11, ^∗^12, ^∗^13) were significantly associated with overall HATT (Supplementary Table S1, http://links.lww.com/MD/A303)

**TABLE 1:**
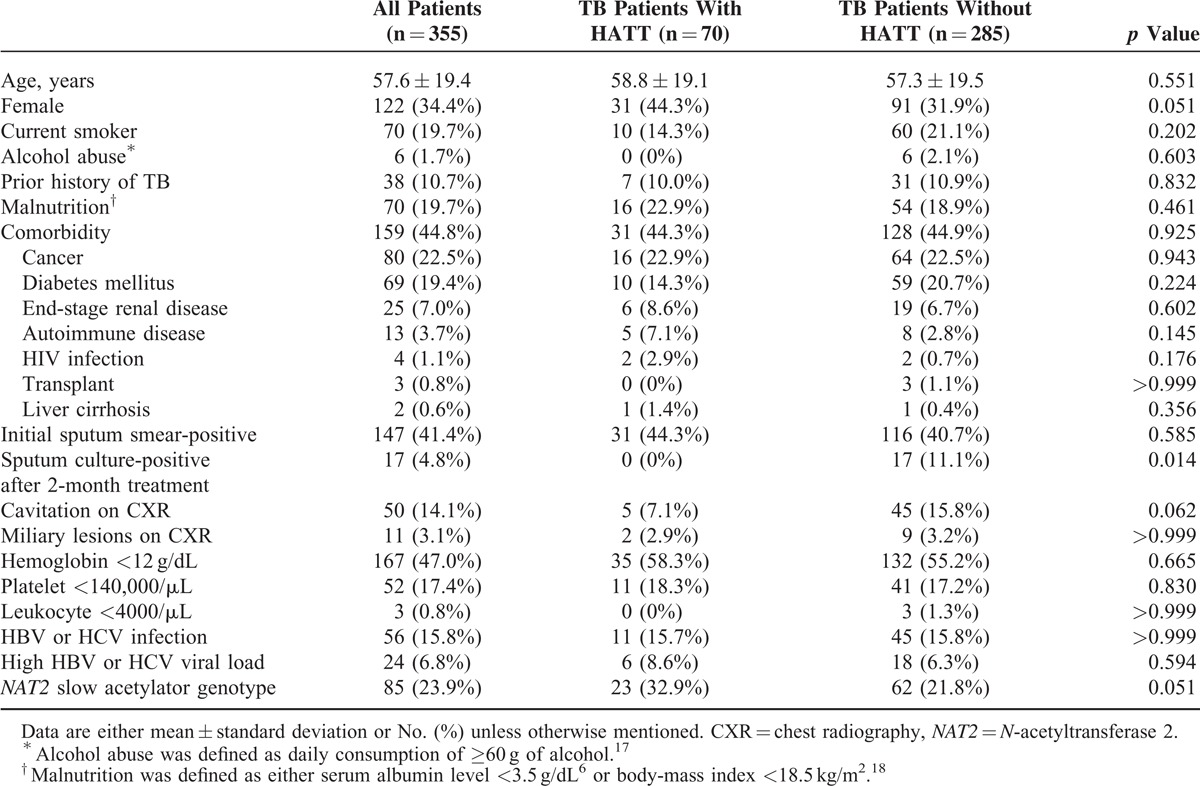
Clinical, Laboratory Characteristics, and *NAT2* Genotype of Tuberculosis (TB) Patients With Drug-Induced Hepatitis During Antituberculous Treatment (HATT) (n = 70) and the TB Patients Without (n = 285)

### Genotype of *PXR* SNPs and Association With Overall Drug-Induced HATT

All of the participants had *PXR* SNPs successfully genotyped, except for 1 man and 1 woman in rs2461823, 1 man in rs7643645, and 2 men in rs12488820 and rs3814057. All *PXR* polymorphisms met the Hardy-Weinberg equilibrium (*P* > 0.05) except for rs12488820 (*p* < 0.001) and rs3814057 (*p* = 0.002). Table 2 shows the risk of drug-induced hepatitis among TB patients with different SNP genotypes in *PXR* gene (see also Supplementary Figure S2, http://links.lww.com/MD/A303). In male TB patients, none of the 6 SNPs in the *PXR* gene was associated with the development of overall drug-induced HATT, with similar genotype frequencies between male patients with hepatitis and those without. In female TB patients, 2 *PXR* variants were significantly associated with the risk of overall drug-induced hepatitis. The frequency of drug-induced HATT in female patients with AA genotype at rs2461823 site (50%) was significantly higher compared to female patients carrying other genotypes (AG, 22%; GG, 15%, *p* = 0.007). The higher risk of AA genotype at rs2461823 in females was also shown in the recessive model (odds ratio [OR] and 95% confidence interval [CI]: 0.24 [0.10–0.63]) and additive model (OR: 2.4 [1.30–4.44]) (Supplementary Table S2, http://links.lww.com/MD/A303). On the other hand, the frequency of HATT in female patients with AA genotype at the rs7643645 site (3%) was significantly lower compared to female patients with other genotypes (AG 30%, GG 37%, *P* = 0.004). The protective effect of AA genotype at rs7643645 in females was also evident in the dominant model (OR: 14.0 [1.82–108]) and additive model (OR: 2.56 [1.37–4.76]) (Supplementary Table S2, http://links.lww.com/MD/A303). Both SNPs were located at intron 1 (Table 2), near the promoter of the *PXR* gene.

**TABLE 2:**
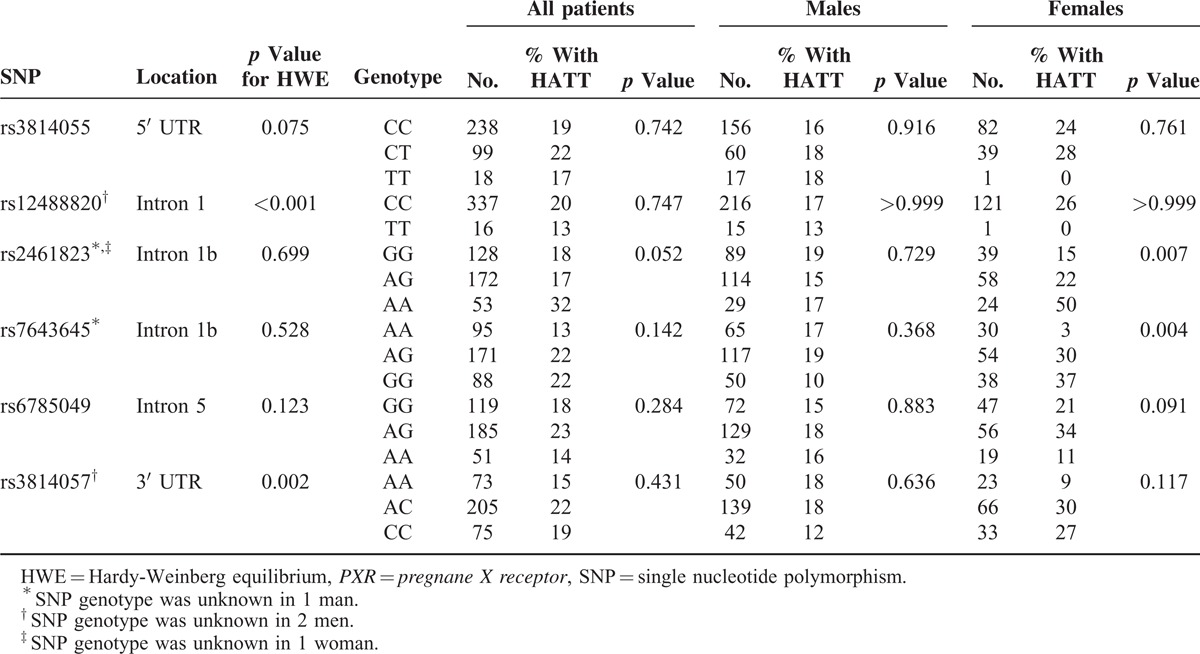
Influence of Genotype at Each *PXR* SNP Site on Drug-Induced Hepatitis During Antituberculous Treatment (HATT) in Males and Females Using Chi-Square Analysis

### Haplotype Frequency and Association With Overall Drug-Induced HATT

There were 7 haplotypes with a frequency ≥0.05 (Supplementary Table S3, http://links.lww.com/MD/A303). In male TB patients, none of the 7 haplotypes were associated with the development of overall drug hepatitis (Table 3). However, in female TB patients, 3 haplotypes, h001101 (OR: 3.08 [1.24–7.61]), h001110 (OR: 3.04 [1.29–7.16]), and h000110 (OR: 2.59 [1.12–5.98]), were significantly associated with increased risk of drug-induced HATT.

**TABLE 3:**
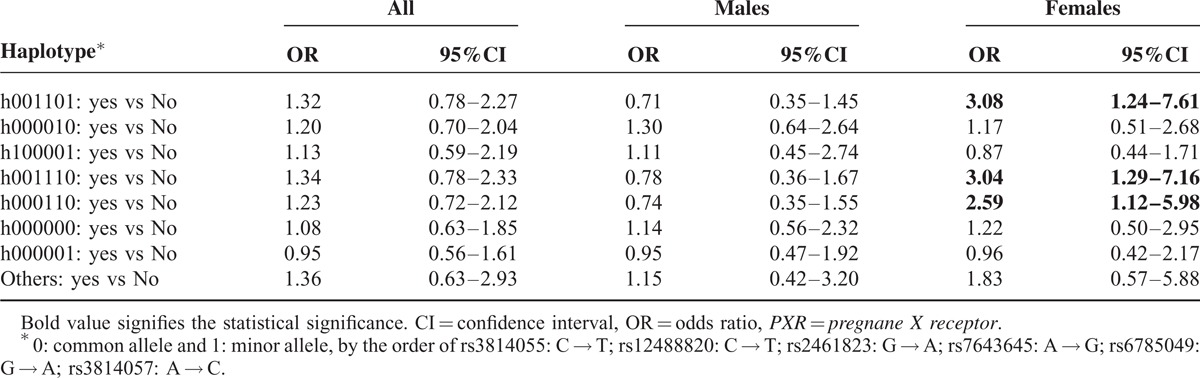
Risk of Drug-Induced Hepatitis During Antituberculous Treatment (HATT) in Males and Females With Different Haplotypes of the *PXR* Gene

### Multivariate Analyses for Risk Factors of Overall Drug-Induced, INH-, RIF-, and PZA-Induced HATT

Multivariate logistic regression analysis, including all the variables listed in Table 1, *NAT2* variants, *PXR* genotypes, allele numbers, haplotypes, and sex interaction, was performed to identify independent predictors of overall and individual drug-induced HATT. Table 4 shows that the only independent predictors of overall drug-induced HATT were *PXR* variants genotypes, allele numbers, and haplotypes. Genotype analysis revealed that the female genotype AA at rs2461823 site (OR 4.64 [1.96–11.0]), a risk genotype, and the female genotype AA at rs7643645 site (OR: 0.14 [0.02–1.02]), a protective genotype, were both independently associated with overall drug-induced HATT. Allele number analysis showed that the number of G allele at rs7643645 was significantly associated with increased risk of drug-induced HATT in females (OR: 1.91 [1.35–2.71]). Haplotype analysis revealed that 2 haplotypes (both carrying G allele at rs7643645 site), h001101 (OR 2.30 [1.22–4.32]) and h000110 (OR 2.25 [1.08–4.69]), were associated with increased risk of overall drug-induced HATT in females.

**TABLE 4:**
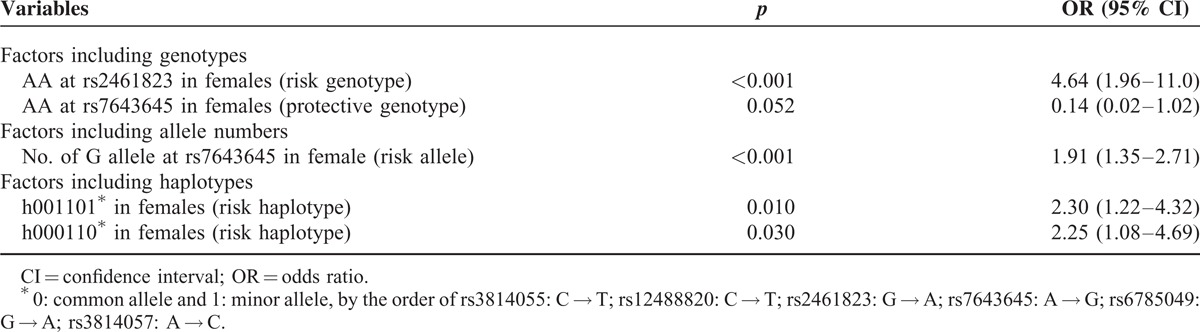
Factors Associated With all Drug-Induced Hepatotoxicity During Antituberculous Treatment, by Multivariate Logistic Regression Analysis

As for INH-induced hepatitis, 1 *NAT2* variant (857G>A, corresponding to *NAT2*^∗^7, a slow acetylator genotype) was associated with increased risk, and the wild type allele *NAT2*^∗^4, a rapid acetylator genotype was associated with decreased risk of INH-induced HATT (Supplementary Table S1, http://links.lww.com/MD/A303). In multivariate logistic regression analysis, *NAT2* and malnutrition were independent risk factors for INH-induced hepatitis in both male and female patients, but genotype AA at rs2461823 site (OR: 10.5 [1.91–58.1]) and number of A allele at rs6785049 site (OR: 11.7 [1.06–129]) were independent risk factors only in females (Supplementary Table S4, http://links.lww.com/MD/A303). None of the *PXR* haplotypes were significantly associated with INH-induced hepatitis.

For RMP-induced hepatitis (Supplementary Table S5, http://links.lww.com/MD/A303), multivariate logistic regression analysis revealed that end-stage renal disease, number of A allele at rs6785049, and h000010 haplotype were independent risk factors in both male and female patients, but genotype AG at rs6785049 (OR: 3.09 [1.09–8.81]) and h001101 haplotype (OR: 5.51 [1.68–18.1]) were independent risk factors only in females.

For PZA-induced hepatitis (Supplementary Table S6, http://links.lww.com/MD/A303), multivariate logistic regression analysis revealed that genotype AG at rs7643645 (OR: 2.85 [1.33–6.11]) was an independent risk factor for both male and female patients, but genotype AA at rs2461823 (OR: 7.29 [2.54–20.9]), number of G allele at rs7643645 (OR: 1.84 [1.19–2.85]), and h000110 haplotype (OR: 5.10 [1.92–13.5]) were independent risk factors only in females.

### Validation for Risk Factors of Overall Drug-Induced HATT

A total of 182 TB patients were enrolled into the validation cohort. Their mean age was 58.3 ± 37.5 years and 114 (62.6%) were male. During follow-up, 18 (26%) of the 68 female patients and 17 (15%) of the 114 male patients developed HATT.

Table 5 shows the influence of genotypes at each *PXR* SNP site on the risk of drug-induced HATT. Although none of the SNP genotypes at rs7643645 or rs2461823 were significantly associated with HATT by chi-square test, risk of HATT was higher in female patients with AA genotype at rs2461823 (56%) and GG genotype at rs7643645 (39%). Table 6 compares risk of drug-induced HATT in patients with or without risk predictors. It is evident that the risk of drug-induced HATT was significantly different between the 3 subgroups with different combination of genotypes at rs7643645 and rs2461823 (*p* = 0.018), borderline different in the 3 subgroups with different number of G allele at rs7643645 (*p* = 0.057), and significantly different in the 4 subgroups with different haplotypes (*p* = 0.008). Although the *p* values in the female patients alone did not reach statistical significance, probably owing to the small sample size of the validation group, these findings suggested that the risk of drug-induced HATT was influenced by above-mentioned *PXR* variants and haplotypes only in females.

**TABLE 5:**
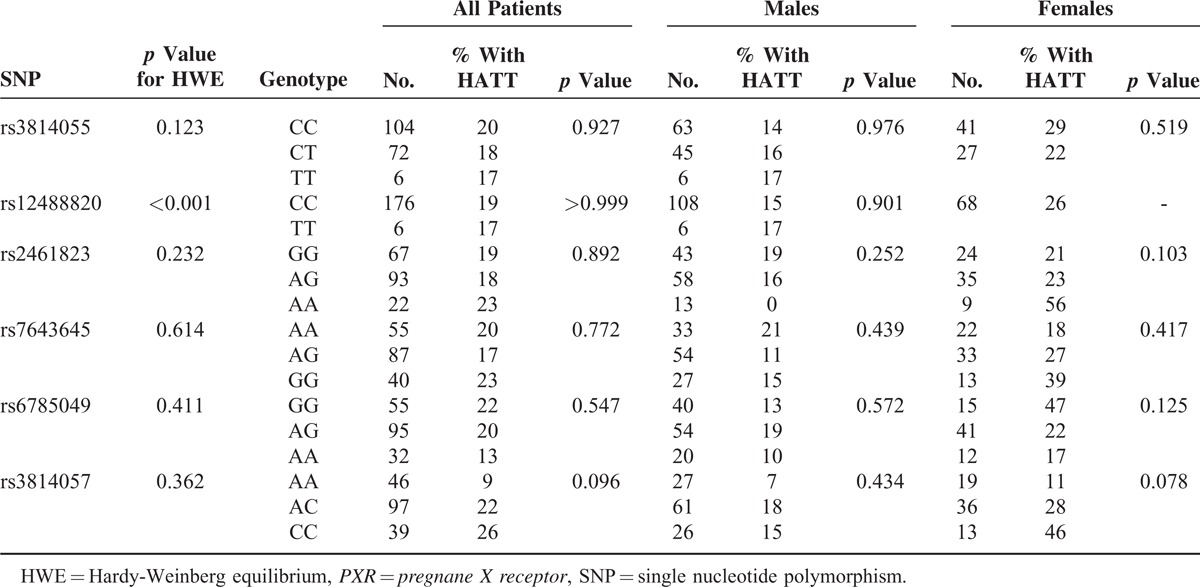
Influence of Genotype at Each *PXR* SNP Site on Drug-Induced Hepatitis During Antituberculous Treatment (HATT) in Males and Females Among Validation Cohort (n = 182) Using Chi-Square Analysis

**TABLE 6:**
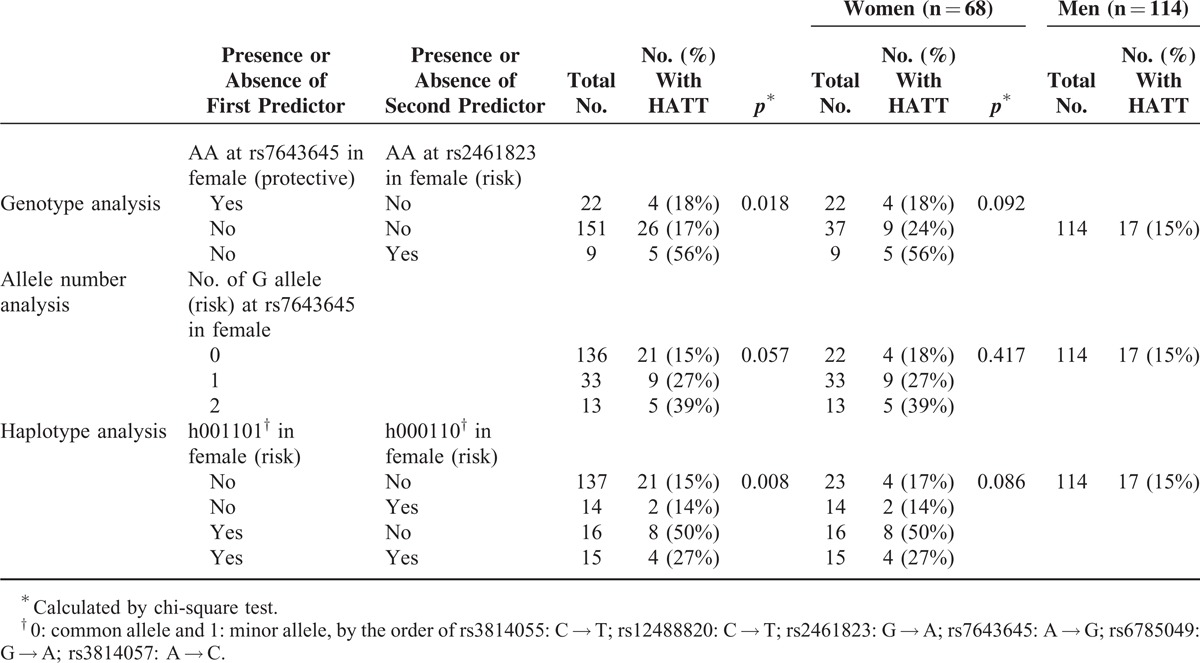
Risk of Drug-Induced Hepatitis During Antituberculous Treatment (HATT) in Validation Cohort (n = 182)

## DISCUSSION

By prospective observation and genotyping in 355 culture-confirmed pulmonary TB patients and validation in another 182 cases, this is the first study to show that the SNPs of the *PXR* gene are independent risk factors of overall drug-induced hepatitis in TB patients, and that the association occurs only in women. Risk factors of hepatitis for individual first-line anti-TB drugs vary, but *PXR* SNP genotypes and haplotypes remain significant risk factors and show gender difference.

Both rs7643645 and rs2461823 were located at intron 1b of the regulatory region close to the *PXR* promoter. Rs7643645 is located in the binding site of the transcription factor, hepatic nuclear factor (HNF)4α, and a change from the wild A allele to the mutant G allele leads to a loss of the HNF4α binding site.^13^ Holloway et al^28^ found that in a mouse model, HNF4α exerted both a positive and a negative regulatory effect on many hepatic genes. With loss of the HNF4α, 82% of the 4994 HNF4α-dependent genes were suppressed in males. In contrast, only 56% of HNF4α-dependent genes were suppressed, while some of the HNF4α-dependent genes were upregulated upon loss of HNF4α in females.^28^ Such findings suggest that *PXR* gene expression (and its regulation on target xenobiotic metabolism genes) in response to HNF4α loss is different between the 2 sexes.

As for rs2461823, there has been no transcription factor binding sequence identified in this site to date. Nonetheless, it is in strong linkage disequilibrium with several other SNP sites in the *PXR* promoter region, including rs2472677, which is located within a hepatocyte nuclear factor 3-β (HNF3β) binding site. Its CC genotype is associated with high level *PXR* induction by RMP.^29^ In mice, the HNF3β binding site has been associated with female predominant expression of the *CYP2b9* gene.^30^ Thus, the rs2461823 AA genotype may occur in strong linkage disequilibrium with certain genotypes of other SNPs (such as rs2472677) that bear a transcription factor binding sequence, and this may become associated with different risk of HATT between males and females.

A previous study found that the rs7643645 GG and rs2461823 AA genotypes were associated with increased risk and/or severity of nonalcoholic fatty liver disease, but without any sex difference.^21^ Another study found that the rs2461823 GG genotype was associated with susceptibility to intrahepatic cholestasis of pregnancy. In that study all of the subjects were females.^29^ These reports, together with the current findings, suggest that these 2 *PXR* SNPs affect detoxification and elimination pathways of drugs like INH and PZA, either in females alone or in both sexes. The underlying mechanisms of these observations may be a loss of *PXR* transcription factor binding site.

The findings that SNP in rs2461823 of the *PXR* gene is associated with both INH- and PZA-induced hepatitis have never been reported. The hepatotoxin that leads to INH-induced hepatitis has been proposed to be hydrazine and other hydrazine metabolites that can generate free radicals. The conversion from INH to hydrazine involves either a classical P450 oxidase that can be induced by PXR ligands like RMP, phenobarbital or dexamethasone,^31^ or an amidase which can also catalyze the reaction from monoacetylhydrazine to hydrazine (Supplementary Figure S3, http://links.lww.com/MD/A303).^32^ Monoacetylhydrazine can be converted to free radical hepatotoxins by a P450-dependent CYP2E1. Therefore, PXR could increase the production of hydrazine, free radical hepatotoxins, and thus the risk of INH-induced hepatitis, through P450 oxidase and CYP2E1. Whether PXR could influence amidase is unknown. In addition, a recent study has demonstrated that cotreatment with INH and RMP causes liver injury through PXR-mediated alteration of the heme biosynthesis pathway.^33^

As for PZA-induced hepatitis, recently PZA metabolites pyrazinoic acid and 5-hydroxypyrazinoic acid have been reported to correlate with PZA-induced hepatitis.^34^ The conversion of PZA to pyrazinoic acid, and that of 5-hydroxy-PZA to 5-hydroxypyrazinoic acid both involve an amidase (Supplementary Figure S4, http://links.lww.com/MD/A303).^35^ The other source of 5-hydroxypyrazinoic acid production is from pyrazinoic acid via xanthine oxidase. There has been no report regarding to the effect of PXR on the activity of amidase or xanthine oxidase. Since both INH-induced and PZA-induced hepatitis were associated with rs2461823 variant, it was also possible that PXR's effect was through its regulation on amidase, which metabolizes both INH and PZA. Further studies are needed to clarify these hypotheses.

The mechanism of hepatotoxicity due to rifampicin and its derivatives remains unknown. It is hydrolyzed in liver by acrylacetamide deacetylase (AADAC; for rodents, Aadac), a member of the carboxylesterase-5 family, which catalyze the hydrolysis of many ester- and amide-containing chemicals.^36^ A previous study in mice revealed that Aadac mRNA was highly expressed in mouse livers and was suppressed by 2 PXR ligands—pregnenolone-16α-carbonitrile and dexamethasone.^37^ However, whether the Aadac mRNA expression was altered by the SNP polymorphisms of the *PXR* gene was not studied. The finding that the 1 genotype and 2 haplotypes of the *PXR* gene were significantly associated with RMP-induced hepatitis in the study suggests that PXR may regulate the metabolism of RMP through AADAC and alter the risk of hepatitis. Further studies are needed to confirm these finding and investigate the underlying mechanisms.

From the results of genotype distribution we calculated the risk of anti-TB drug-induced HATT associated with haplotypes, and found that 2 haplotypes were also associated with the risk of HATT by multivariate analysis. We observed that all the 3 *PXR* SNPs (rs2461823, rs7643645, and rs6785049) contributed to the association between haplotypes and HATT.

We also observed that NAT2 slow acetylator and malnutrition were independent risk factors for INH-induced hepatitis both in males and females, consistent with previous reports.^6,38^ Malnutrition has been reported to reduce the activity of hepatic glutathione S-transferase and increase vulnerability to oxidative injury,^39^ leading to increased risk of INH-induced hepatotoxicity in TB patients.

The finding that end-stage renal disease is associated with RMP-induced hepatotoxicity is unexpected since 60% to 65% of RMP dose appears in feces, and it does not accumulate in patients with impaired renal function (Rifadin package insert, Marion Merrell Dow, Ohio, US). Because uremic toxins can alter the hepatic clearance of many drugs, either by downregulation of specific isoforms of CYP via affecting promoter^40^ or by impaired hepatic uptake mediated by uptake transporters,^41^ it is possible that the increased risk of RMP-induced hepatotoxicity in patients with end-stage renal disease was associated with the accumulation of uremic toxin.

The present study has limitations regarding the interpretation of its findings. First, the mechanisms of INH-, RMP-, or PZA-induced HATT are likely to be different, and mixing together all patients with drug-induced HATT may mask unknown risk factors. Yet we analyzed risk factors for individual drugs and found that risk factors for individual drugs also included *PXR* variants. Second, drug-metabolizing enzymes other than PXR and NAT2 were not genotyped. Third, serum levels of drugs and toxic metabolites were not measured. Nonetheless, even if serum levels of metabolites have been shown to be highly predictive for some of HATT, routine measurement is not practical. Fourth, the validation cohort is small in sample size and did not directly replicate the results of the derivation cohort. Yet when we further analyzed patients with the presence or absence of the risk factors that were identified in the derivation cohort, the influence of *PXR* variants and haplotypes was still evident in the female patients of the validation cohort. Lastly, the study was conducted in a medical center and nearly half of the TB patients had underlying comorbidities that might influence laboratory results and radiographic findings.

## CONCLUSIONS

This study is the first to show that 2 *PXR* SNP genotypes and 2 haplotypes influenced the risk of HATT only in females. The *PXR* gene variants have sex-dimorphic impact that contributes to the increased risk of drug-induced HATT in females.
